# 3D-Printed Microrobots: Translational Challenges

**DOI:** 10.3390/mi14061099

**Published:** 2023-05-23

**Authors:** Misagh Rezapour Sarabi, Ahmet Agah Karagoz, Ali K. Yetisen, Savas Tasoglu

**Affiliations:** 1 School of Biomedical Sciences and Engineering, Koç University, Istanbul 34450, Türkiye; msarabi19@ku.edu.tr (M.R.S.); akaragoz22@ku.edu.tr (A.A.K.); 2 Koç University Is Bank Artificial Intelligence Lab (KUIS AI Lab), Koç University, Istanbul 34450, Türkiye; 3 Department of Chemical Engineering, Imperial College London, London SW7 2AZ, UK; a.yetisen@imperial.ac.uk; 4 School of Mechanical Engineering, Koç University, Istanbul 34450, Türkiye; 5 Koç University Translational Medicine Research Center (KUTTAM), Koç University, Istanbul 34450, Türkiye; 6 Koç University Arçelik Research Center for Creative Industries (KUAR), Koç University, Istanbul 34450, Türkiye; 7 Boğaziçi Institute of Biomedical Engineering, Boğaziçi University, Istanbul 34684, Türkiye

**Keywords:** microrobots, 3D printing, clinical translation, biomaterials

## Abstract

The science of microrobots is accelerating towards the creation of new functionalities for biomedical applications such as targeted delivery of agents, surgical procedures, tracking and imaging, and sensing. Using magnetic properties to control the motion of microrobots for these applications is emerging. Here, 3D printing methods are introduced for the fabrication of microrobots and their future perspectives are discussed to elucidate the path for enabling their clinical translation.

## 1. Three-Dimensional Printing for the Fabrication of Microrobots

Three-dimensional printing is an additive manufacturing process that uses sequential layer-by-layer material deposition and digital control with predefined computer-aided design (CAD) models to build 3D structures [1,2]. Three-dimensional printing has constantly evolved to create complex 3D structures with design flexibility, significant material reductions, and optimal manufacturing durations since its inception in the 1980s as the stereolithography (SLA) method [3]. Three-dimensional printing provides several advantages over traditional manufacturing methods. For example, its flexibility results in the optimal use of energy and resources to minimize production expenses. Furthermore, rapid prototyping using 3D printing provides early identification of design/manufacturing faults, preventing high-cost and time-consuming repairs at later stages [4,5]. Three-dimensional printing also enables customized accurate multi-material production with high reproducibility, design flexibility, and automation, with predefined qualities and functionalities [6]. Various technologies of 3D printing such as SLA, two- or multi-photon lithography (TPP/MPP), direct laser writing (DLW), and fused deposition modeling (FDM) have been utilized for the fabrication of microrobots with the ability to systemically integrate unique structures, components, properties, and functionalities into autonomous 3D micromachines via a single-step, on-demand flexible production method.

**Figure 1:**
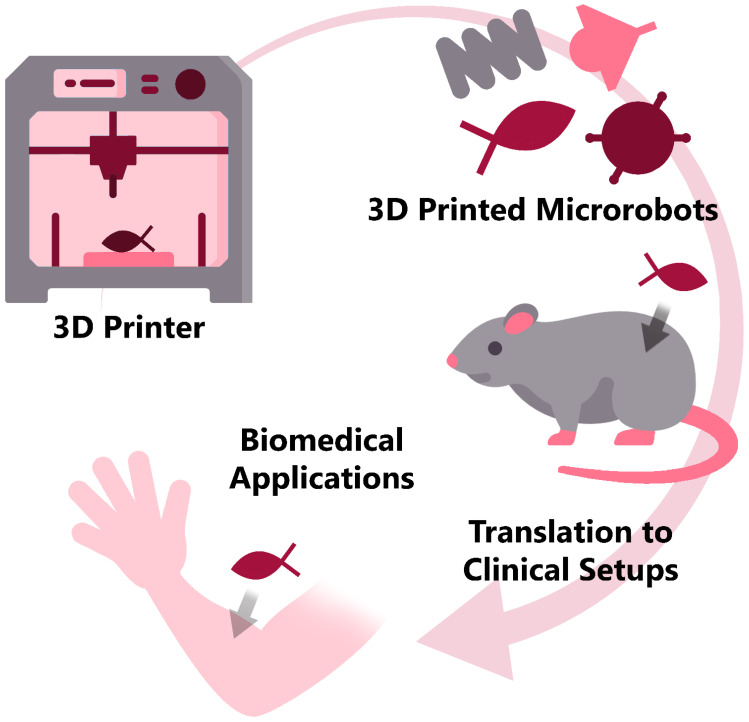
Three-dimensional printing technology provides an effective means for simple, low-cost, and reproducible fabrication of different microrobot types with diverse designs and high-end biomedical applications, such as targeted drug delivery, advanced surgical procedures, particle monitoring, tissue regeneration, and biosensing toward translational medicine.

The field of microrobotics aims to achieve functions such as navigation in complex media, response to their surroundings, and self-organization in sub-millimeter mobile robotic devices [7]. Microrobots can be fabricated in a variety of shapes, featuring diverse types of movements, and functions. For instance, microswimmers, microbowls (i.e., bowl-structured microrobots), microrockets (i.e., bubble-propelled tubular micromachines), microwheels (i.e., wheel-shaped rolling structures), microcarriers, and microdrillers are commonly employed for microscale operations and drug-delivery purposes [2]. On the other hand, microspinners, microdaggers, microrollers, and microfloaters are used to perform single-cell surgery and drug release. For particle manipulation and lab-on-chip mixing, microkayaks (i.e., double cone-rod kayak-paddle shaped microrobots), and microgrippers are preferred. Microcannons (i.e., hollow conically-shaped structures capable of loading and firing nano- and micro-bullets) are the microscale ballistic devices of choice for cargo loading/firing and targeted penetration/deformation in deep tissues. Furthermore, microrafts (i.e., structures made up of spinning circular magnetic structures with sinusoidal edge-height profiles at the air–water interface) are used for dynamic and programmable self-assembly and micro-object manipulation. Additionally, micromotors, microengines, micropumps, and micropropellers are ideal choices for delivery and transportation of specific payloads [2]. In general, depending on their end use, microrobots can be designed and functionalized accordingly for scientific and industrial applications. Three-dimensional printing technology provides cost-efficient fabrication of different microrobot types with diverse designs and high-end biomedical applications toward translational medicine (Figure 1).

Magnetic soft microrobots have the advantages of magnetically driven components with penetration capabilities and modest adverse reactions. Since most of the soft materials are non-magnetic, magnetic powder is externally added to them to drive soft microrobots with magnetic power. While most of the research on magnetically driven soft robots has been carried out on their motion mechanisms and functional shapes, their manufacturing methods have received less attention. One study on this aspect applied an auxiliary grid method to manufacture soft microrobots in a highly controllable and flexible way with a micro 3D-printing system [8]. The auxiliary grid method divides the entire printing area into small grids; hence, different printing materials and printing parameters can be used while printing each consecutive grid. Furthermore, adding silicon to a specific area allowed for printing two-layer structures created from different materials, as a separate material can be added on top of intentionally short-printed material. Additionally, the auxiliary grid method provided the ability to print thinner and softer microrobots, enhancing the deformability of the robots by changing the surface tension. The grid-assisted 3D-printing method not only enabled the printing of soft microrobots made of different materials, but also increased the ability to control the robot’s shape by changing the grid thickness as needed, resulting in high flexibility.

Utilizing the TPP 3D-printing method, microrobots can also be created in detailed microscale 3D shapes for biomedical applications. In this regard, real-time tracking and identification of TPP-based 3D-printed helical microswimmers with half-cylindrical head and helical tail and rectangular control platforms were implemented using different color expression patterns [9]. The fabrication of structurally colorized microrobots started with depositing IP-DIP photoresist on a glass slide and printing the microrobots with TPP, followed by the deposition of Ni and Ge onto the printed structures which were cleaned up from unpolymerized photoresist, and finalized by transporting microrobots to aqueous medium and releasing them with a micropipette. A structural colorization method was used for real-time tracking in which information was obtained not only about the location and identities of the microrobots, but also about their orientation, as the structurally colored microrobots expressed different color patterns at different rotation angles with respect to the light source. Experiments were conducted on helical microrobots to observe color patterns in visible light by applying two coating compositions at different rotation angles and vertical tilts, with different structural color-tracking point designs on silicon wafer substrates as background. The geometric properties of the structural color tracking points were only dependent on the limits of the 3D printer.

Microscale thermal and electrostatic actuators were fabricated using the TPP method, using IP-S negative photoresist and Al sputtering, eliminating the need for additional steps to electrically isolate the actuators from the substrate [10]. Furthermore, 3D printing was used to create electrically-isolated actuators in hanging structures that shadowed a part of the structure. Through this method, a two-beam thermal actuator was produced with a maximum deflection of 18 μm upon application of 8 mA current. On the other hand, side paneling has been used to create an electrostatic actuator with alternating comb structures. Their deflection after applying an AC voltage (50–160 V) was measured with a maximum value of 12.7 μm at 160 V, which shows a linear relationship between the deflection and the voltage increase. Furthermore, the proposed method produced thermally corrugated wing-like structures with a length of 500 μm, for which a displacement of 18 μm was recorded at an excitation of 8 mA. Additionally, when the wings were subjected to a cyclic test of 8 mA and 10 Hz, they successfully endured 8500 cycles. The electric potential applied to the actuators caused an upward expansion, which resulted in the rotation of the wingtips. The amount of the deflection at the wing tip upon application of 2 V (peak-to-peak), 1 Hz square electrical potential was analyzed by optical imaging, where the maximum displacements in -x and -y directions were 15 μm and 5.5 μm, respectively, indicating the potential of 3D-printed microstructures to be employed as micro-actuators in microrobots.

SLA 3D printing has also been reported for the fabrication of microrobots. In this context, resin-based, magnetically moveable scaffolds were made using the SLA approach in conjunction with electroless metallization (length: 4.5 mm and 3 mm) with cell-carrying ability [11]. A Cu layer (400 nm) was coated onto printed structures to increase the adhesion of the following layer to the structure. In addition, 4.5 µm-thick CoNiP films were grown on the structures to facilitate semi-hard-magnetic features. The final structure of the devices was formed by subsequent coatings of NiP (1.1 µm) and Au (80–120 nm) layers. The Au layer was used to enhance the biocompatibility and integrity of the devices, while the NiP layer was used to increase the ability of the devices to be coated with Au. The scaffolds were submerged in water and oil, subjected to RMFs, and tested for movability. The linear speed of the scaffolds increased by an increase in magnetic field frequency in water and air, while the speed was limited to roughly 1 mm·s^−1^ (frequencies > 0.8 Hz) in oil. Inspecting the scaffolds’ impact on cell viability revealed 90% cell vitality for cells exposed to Au-coated scaffolds after two days. This method prospected a cost-effective manufacture of microscaffolds that were potent for targeted cell transport in the human body, such as in the gastrointestinal tract or large vessels.

**Figure 2:**
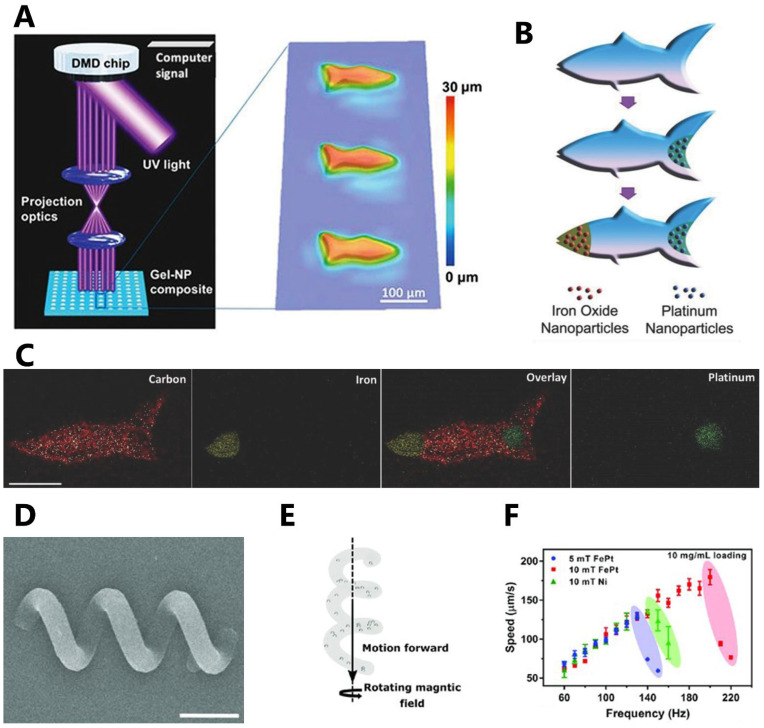
Three-dimensionally printed microrobot examples. (**A**) Hydrogel microfish with biomimetic structures and locomotive ability, along with functionalized nanoparticles, were developed using microscale continuous optical printing (μCOP). (**B**) Schematics of functionalizing a microfish for guided catalytic propulsion. Pt nanoparticles were loaded into the tail of the fish for propulsion, and Fe_3_O_4_ nanoparticles were loaded into the fish head for magnetic control. (**C**) Energy-dispersive X-ray spectroscopy illustrated the iron-oxide head and platinum tail with respect to the PEGDA microfish body (scale bar: 50 μm). (**D**) SEM image of 3D-printed magnetic helical microswimmer fabricated using two-photon polymerization (TPP) (scale bar: 10 μm). (**E**) The velocity vectors of the microswimmers with forward and rolling velocity components. (**F**) Swimming performance evaluation results for various loadings of FePt. Subfigures (**A**–**C**) were adapted with permission from [12], and subfigures (**D**–**F**) were adapted from [13] in accordance with the CC-BY license.

A hydrogel artificial fish was developed with propulsion achieved through catalytic impulsion using microscale continuous optical printing (μCOP) method (Figure 2A–C) [12]. The micro-robotic fish had biomimetic structure and locomotive ability. The fish’s tail was loaded with functionalized platinum (Pt) nanoparticles using catalytic decomposition, enabling propulsion. Additionally, the fish’s head was loaded with Fe_3_O_4_ nanoparticles to facilitate magnetic actuation and control. Energy-dispersive X-ray (EDX) spectroscopy images showcased the microfish’s body made of poly(ethylene glycol) diacrylate (PEGDA), the platinum tail, and the iron-oxide head. Another study developed magnetic helical microswimmers using TPP 3D printing (Figure 2D–F) [13]. The design featured a trimethylolpropane ethoxylate triacrylate (PETA) magnetic helix with three turns, incorporating either FePt or superparamagnetic iron-oxide nanoparticles (SPIONs). The swimming velocity of the microswimmers, measuring 30 μm in length, was evaluated. It was observed that the FePt-based microswimmers exhibited higher endurance at higher frequencies, resulting in higher velocities compared to the other microswimmers. It was also found through *in vitro* experimentation that the synthesized FePt nanoparticles were biocompatible. Presenting a simple actuation method, these microswimmers can enable the creation of clinically viable future medical microrobots.

Four-dimensional printing, introducing the “time” parameter into the 3D-printing fabrication procedure [14] (i.e., forming structures that can change their shapes or functionalities after the printing procedure, using external stimuli such as magnetic field, electricity, light, pH, or temperature), is emerging for the manufacturing of microrobots. A biodegradable artificial bacterial flagella (ABFs) was developed through a DLW 3D printer and functionalization of photocurable gelatin with acrylic groups [15]. Nevertheless, adding functional groups (such as acrylates) significantly reduces biocompatibility. This led to the suggestion of an indirect way to produce 3D-printed sacrificial templates using DLW’s high-resolution micromolds. With a minimum dimension of 5 µm, the discovered approach enabled the manufacturing of 4D stent-like microstructures. The 4D printing process made use of a shape memory polymer (SMP). Since it is still difficult to find the right mix of photo-initiators and monomers to create biocompatible SMPs with operating temperatures in the range of body temperature, SMPs could not be produced using DLW. The proposed microstent had a Young’s modulus in the order of 1 GPa, similar to that of commercial polymeric medical stents, while the reported Young’s modulus for the 4D-printed stent is about 100 MPa. The 3D shape of the sacrificial templates was produced by spin coating followed by the soft-bake process. This method to fabricate soft microrobots demonstrated an indirect printing strategy, which can elucidate the path for 3D- and 4D-printed soft microrobots for future medical setups.

## 2. Outlook and Challenges in Clinical Translation

The clinical use of microrobots is facing several scientific and technical challenges: microrobots should not only be able to overcome biological barriers of the body but they should also possess biocompatibility and the capability of functioning in complex biological fluids, as well as preferable biodegradation propensity [16]. Furthermore, from the preclinical stage to bedside application of microrobots, all safety aspects and challenges need to be considered. For determining the regulatory pathways for microrobots, it is first necessary to decide whether the proposed microrobots are categorized as a drug or as a medical device, since different rules apply for each category [16]. Since control techniques, objectives, physical designs, and configurations of microrobots are application-specific, each microrobot should be evaluated separately.

Biodegradability is beneficial in biomedical microrobots for several reasons; first, biodegradable microrobots minimize the risk of long-term adverse effects or complications when introduced into the body. Once their intended purpose is fulfilled, they can naturally break down and be eliminated, reducing the potential for toxic accumulation. Second, biodegradable microrobots eliminate the need for additional invasive procedures or interventions to retrieve them from the body. As they degrade over time, there is no need for surgical removal, reducing patient discomfort and complications. Third, biodegradable materials used in microrobots are designed to be compatible with biological systems, minimizing potential immune reactions or rejection responses from the body. This ensures that the microrobots can perform their intended functions without causing harm or discomfort. Finally, biodegradable microrobots have a reduced environmental impact compared to non-biodegradable counterparts. After their useful lifespan, they can degrade naturally without leaving behind non-biodegradable waste. Overall, biodegradability in biomedical microrobots enhances safety, compatibility, and convenience, making them more suitable for a wide range of applications in medicine and healthcare.

**Figure 3:**
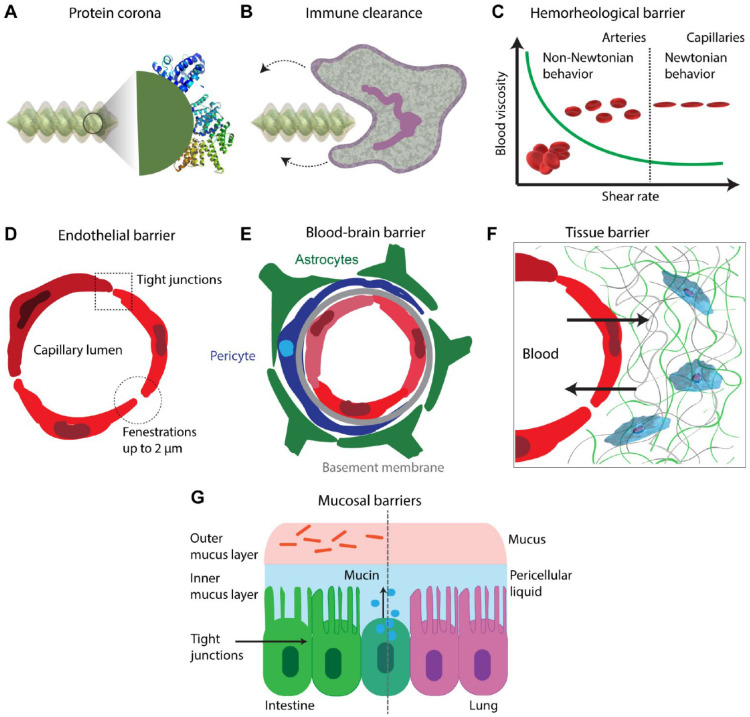
Potential biological barriers for administration of microrobots and/or enabling their functionality. (**A**) Formation of protein corona on the microrobots’ surfaces. (**B**) Immune clearance by phagocytes caused by connection of opsonin proteins. (**C**) Hemorheological barrier and obstacles caused by the blood flow behavior. (**D**) Endothelial barrier and other challenges in cellular level. (**E**) Blood–brain barrier (BBB). (**F**) Fibrous extracellular matrix (ECM) of the tissues and high interstitial pressure in tumors. (**G**) Mucosal barrier. Reproduced from ref. [16] in accordance with the CC-BY license.

Microrobots’ *in vivo* expedition from the starting point to the target action zone is fraught with threatening menaces of complex biological barriers (Figure 3) [16]. An adsorption layer, called the protein corona, is usually formed around a microrobot inside the body as a result of an immoderate amount of protein interacting with its surface. As a result of this layer, some functions of the microrobots related to the surface properties are subject to change. First, the swimming speed of the microrobot can be highly influenced by changes in surface polarity. Furthermore, a bigger nuisance is that corona formation may activate protein misfolding and aggregation, creating an immune reaction that can result in the destruction of microrobots. Finally, unforeseen changes in the surface may prevent chemical, biological, or mechanical functions, such as the mechanics of drug release and the sensing mechanism of environmental signals, to act as designed. Additionally, microrobots that are aimed at use in the vascular system may encounter a dynamic rheological imbalance problem of the blood flow, depending on the vessel thickness, stream power, and hematocrit value. Blood is a non-Newtonian fluid [17], and it undergoes a reduction in viscosity as the red blood cells change their main axis to line up with the flow at high shear levels. Thus, microrobots for vascular system applications should be designed and fabricated with properties that can adapt to changes in blood viscosity and flow characteristics. Alternatively, when a microrobot has to pass through extremely small spaces such as the endothelial barrier, the size factor becomes a crucial parameter. For example, the blood–brain barrier (BBB) opening allows for a crossing area smaller than one nanometer. To pass through such tight barriers, a focused ultrasound system (FUS) can be applied on the microrobots to transiently widen tight junctions [18]. Although this system can usually create openings at nanoscale, these openings can be brought to a size that allows microrobots to pass through using a leukocyte extravasation strategy. Another alternative route to the brain is through the cerebrospinal canal. A robot in this path has to deal with protein corona formation, the cerebrospinal fluid (CSF) dynamics problem, immune cell attacks, ependymal layer, and pia mater to reach the brain. Similarly, to reach tumor tissue using the bloodstream, a microrobot will face protein corona, opsonization, immune attack, flow/rheological obstacles, endothelium barriers, intertumoral pressure, and fibrous matrix. Other barriers, such as the interstitial fluid (ISF) pressure and fibrous elements of the tissue extracellular matrix (ECM) are also among the hurdles that microrobots have to overcome. Additionally, one final check before exposing medical devices to human body for their successful clinical translation is an appropriate sterilization procedure, which will prevent the transmission of possible pathogens present on the devices or in the environment to the patient [19,20].

Biocompatibility is a key requirement of the materials used in medical devices, such that their contact with biological structures such as tissues and organs will not cause adverse host responses or trigger immune reaction [21,22,23,24]. As a result, exploring the material engineering aspects of microrobots can ease the clinical translation challenges. One study investigated the material properties and the cytotoxicity effects of magnetic polymer composite (MPC) structures, made out of SU-8 photoresist and magnetite (Fe_3_O_4_) nanoparticles with various particle concentrations, and also explored their potential applications on helical microrobots that were 3D-printed with the TPP technique [25]. MPC microdevices could be constructed to be biocompatible and non-hazardous to cells, and could provide the necessary mobility in the biomedical field when produced in appropriate particle ratios. When MPC microdevices contain particles up to 10% volume, they do not cause significant cytotoxicity within 24 h of incubation, and if they contain particles up to 2% by volume, they can provide the necessary mobility via corkscrew action in water even in weak magnetic fields.

The interaction between the microrobot and the immune system is one of the biggest obstacles for long-term use of microrobots in medical applications. With the new material solutions introduced by zwitterionic photoresists, problems such as biocompatibility and immunogenicity which are faced by microrobots can be solved. For example, zwitterionic non-immunogenic materials were developed to prevent microrobots from being detected and ingested (i.e., phagocytosis) by macrophages [26]. Fully zwitterionic photoresists were used in TPP 3D printing of hydrogel microrobots. The interactions of their zwitterionic microrobots with macrophages were examined by *in vitro* analysis, where the microrobots could not be detected although they interacted with macrophages for a long time (>7 days). Because of the complexity and variability of the immune system, investigations focused only on a specific subsystem. Since macrophages are one of the first obstacles foreign substances encounter in the body, the experiments focused on the interactions of microrobots with macrophages. Particularly, zwitterionic microrobots were not attacked by macrophages because they were not perceived as an external hazard. The reported zwitterionic materials are promising for providing biocompatibility. They can also present the building blocks of non-immunogenic materials in 3D microprinters. In particular, they can be beneficial for the implementations of micromechanisms such as medical microrobots in the bioengineering and biomedical fields.

## 3. Future Perspectives by Integration with Artificial Intelligence (AI)

**Figure 4:**
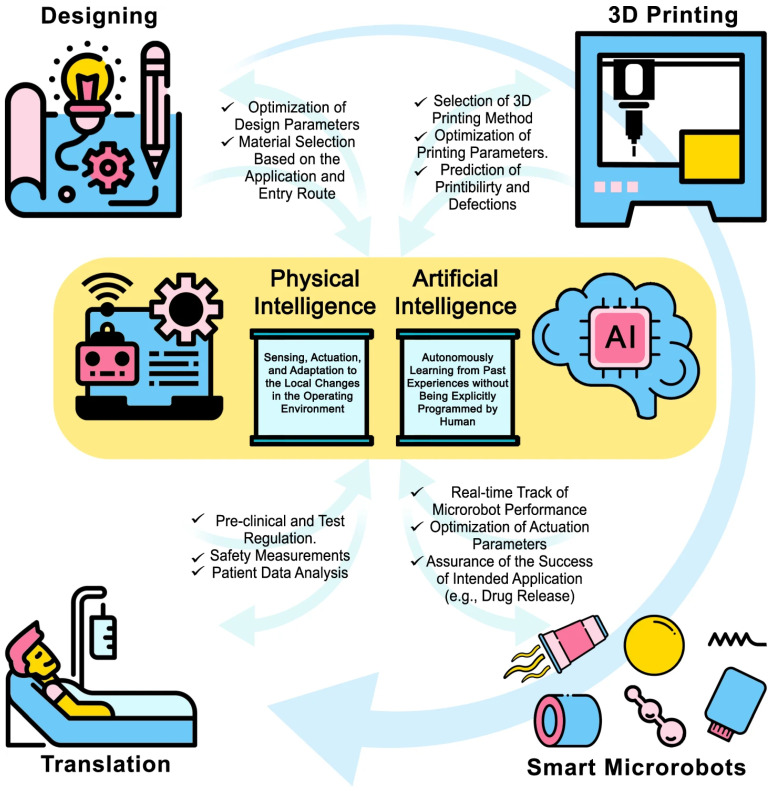
Three-dimensionally printed microrobots from design to translation, along with integration of microrobots with artificial intelligence (AI) and physical intelligence (PI) technologies. Application of AI and PI can aid in designing application-specific microrobots and optimize the 3D printing process to reduce defects. During the application phase, these technologies can assist clinicians in tracking microrobots, improving maneuverability through parameter adjustments, sensing and independently responding to environmental stimuli, such as releasing drugs at specific pH levels. Finally, patient data can be analyzed in a time-efficient manner for personalized recommendations. Adapted from ref. [7] in accordance with the CC-BY license.

Artificial intelligence (AI) is one of the trailblazers for increasing the availability of information about patterns in healthcare data via prompt analysis and comparison along with enhancing rapid progress of analytical methods [27]. Machine learning (ML) is a subfield of AI that focuses on leveraging data and algorithms to mimic human learning. ML can be utilized in a wide variety of sectors such as defect detection in manufacturing processes, sale forecasting in marketing and commercialization steps, and patient data analysis in clinical administration. AI and physical intelligence (PI) can be integrated with microrobots for development of advanced healthcare systems (Figure 4). Various AI techniques, such as support vector machines, multilayer perceptron, and recurrent neural networks, have the ability to make an impact on microrobot applications as well in different stages of their biomedical usage such as detection and diagnosis techniques and treatment procedures, as well as prediction of results and prognosis evaluation. For example, optical interferometry was used for force estimations on microrobots fabricated with TPP by training artificial neural networks, a subset of ML and Deep Learning (DL) algorithms, on high-dimensional spectral measurements to demonstrate force-sensing capability [28]. Furthermore, using vast amounts of data collected from the human body by microrobots, personalized medical microrobots can be created for patient-specific clinical studies and individualized treatments. The utilization of AI techniques can also enable the ability of microrobots to adjust their motion and driving parameters through the information collected from their surrounding environment such as chemical composition, pH, fluid viscosity, and velocity. A significant challenge for microrobots is to overcome numerous biological hurdles to reach the desired destination. Key decisions have to be made to find an optimal path from starting point to destination area in a dynamic environment. The safest and fastest route for the microrobot can be dynamically calculated by DL algorithms, usually with a reinforcement learning technique, which is already utilized on real-time unmanned aerial vehicle (UAV) navigation [29], path planning of mobile robots [30] and autonomous microrobot swarm navigation [31]. In this regard, DL techniques can be helpful solutions not only for detecting and avoiding the obstacles that microrobots encounter as they move through the body, but also reacting through dynamic generation of new paths to avoid any possible collision.

While designing 3D microrobots, several characteristics such as interactions with various chemicals, printability of the overall structure, and functionality in different environments should be considered. Additionally, based on their applications, microrobots should be in specific shapes to fulfill their designed functions. AI methods can be helpful to control and optimize all these design aspects. For example, DL is widely used to generate 2/3D structures in different domains such as designing molecular structures, mechanical models, and protein folding [32]. Using the information of environmental constrains, structural needs, type and duration of the mission, possible obstacles, and chemicals to be interacted, a DL model such as generative adversarial network (GAN) can generate 3D CAD models of a microrobot with material and manufacturing method suggestions. For example, a trained GAN can generate a wisely-designed actuator for fast-moving microrobots or extra carriage module for cargo delivery purposes. Upon designing, the next possible limit can be their manufacturability. There are advanced 3D printing methods that are precise and capable of producing microscale structures; however, they have many parameters that need to be well adjusted for high-quality manufacturing. AI can play a role in optimizing 3D printer parameters as well. For example, ML and DL techniques have been used for anomaly detection and quality prediction of 3D-printed microneedles [33], as well as the optimization of process and material parameters of 3D bioprinters [34]. Additionally, microrobots can be monitored on a continuous basis during *in vivo* medical interventions using medical imaging systems for real-time device tracking, guidance, and localization based on DL techniques for computer vision applications such as object tracking, segmentation, and detection.

## 4. Conclusions

Microrobots have attracted attention owing to their particular properties to accomplish tasks in hard-to-reach sites in the human body. Their biomedical applications are growing to include sectors such as drug delivery, minimally-invasive surgery, biosensing, tissue regeneration, imaging, and particle tracking and monitoring. Three-dimensional printing has emerged for time- and cost-efficient fabrication of microrobots, enabling their rapid growth for potential translational stages. Safety aspects and regulatory pathways should be considered from the preclinical stage to the application of microrobots in a clinical setting. The clinical use of microrobots on the other hand faces several technical challenges related to overcoming biological barriers, biocompatibility, functioning in complex biological fluids, and biodegradation propensity. Material engineering aspects, such as exploring advanced biocompatible materials, can address challenges related to cytotoxicity, immunogenicity, and interactions with the immune system, enhancing the clinical translation of microrobots. AI and PI techniques can also play a critical role in tackling current challenges and improving the functionality of these medical devices. AI and PI can also aid in designing application-specific devices and optimize the 3D printing process to reduce defects. In a nutshell, microrobots will allow for pioneering the development of advanced healthcare systems in personalized medicine.
